# Supplementary material for: A Critical View of the Application of the APEX Software (Aqueous Photochemistry of Environmentally-Occurring Xenobiotics) to Predict Photoreaction Kinetics in Surface Freshwaters
Source: Molecules. 2019 Dec 18;25(1):9. doi: 10.3390/molecules25010009 (PMC7017383; doi:10.3390/molecules25010009)
Supplement: Supplementary file 1 [file molecules-25-00009-s001.zip › 3.2.4_gcc-4.4.0.zip!/3.2.4_gcc-4.4.0/3.2.4_gcc-4.4.0/doc/octave/html/interpreter/Group-Database-Functions.html]

Group Database Functions - Untitled


Next: System Information,
Previous: Password Database Functions,
Up: System Utilities


---

### 34.10 Group Database Functions

Octave's group database functions return information in a structure
with the following fields.

`name`: The user name. `passwd`: The encrypted password, if available. `gid`: The numeric group id. `mem`: The members of the group.

In the descriptions of the following functions, this data structure is
referred to as a grp\_struct.

— Loadable Function: grp\_struct = **getgrent** ()  
> Return an entry from the group database, opening it if necessary.
> Once the end of the data has been reached, `getgrent` returns 0.

— Loadable Function: grp\_struct = **getgrgid** (gid).  
> Return the first entry from the group database with the group ID
> gid. If the group ID does not exist in the database,
> `getgrgid` returns 0.

— Loadable Function: grp\_struct = **getgrnam** (name)  
> Return the first entry from the group database with the group name
> name. If the group name does not exist in the database,
> `getgrnam` returns 0.

— Loadable Function: **setgrent** ()  
> Return the internal pointer to the beginning of the group database.

— Loadable Function: **endgrent** ()  
> Close the group database.
